# Supplementary material for: DNA Barcoding of Chironomid Larvae (Diptera: Chironomidae) from Large Rivers in South Korea to Facilitate Freshwater Biomonitoring and Public Health Surveillance
Source: Int J Environ Res Public Health. 2022 Sep 23;19(19):12035. doi: 10.3390/ijerph191912035 (PMC9566745; doi:10.3390/ijerph191912035)
Supplement: Supplementary file 1 [file ijerph-19-12035-s001.zip › ijerph-1874050-supplementary.pdf]

Table S1. Taxonomic key to species of Chironomidae larvae in large South Korean rivers.

1. Head capsule oval, elongated. Eye spots single, round or kidney-shaped. Ligula strongly and distinctively dentate ....(Tanypodinae) ***Procladius choreus***

1′ Head short. Eye spots more than 2. Ligula not dentate 2

2(1′) Eye spots 2, different in size, arranged horizontally. Some species with 1 eye-spot, shaped like a comma. Mentum with smaller, irrigular-shaped ventromental plate.........................(Orthocladiinae) 3

2′ Eye-spots 2 or 3, similar in size, arranged vertically. Mentum with well developed rectangular- or fan-shaped ventromental plate. .... (Chironominae) 4

3(2) Antennae 4-segmented. Mentum with bumphy, irregular median tooth.......... ***Propsilocerus akamusi***

3′ Antennae 6-segmented. Mentum with 2 pairs of median teeth and 6 pairs of lateral teeth .... ***Hydrobaenus kondoi***

4(2′) Antennae 5-segmented, with characteristic lauterbone organ. Ventromental plates broad.. (Tanytarsini) 5

4′ Antennae 5–8-segmented, with sometimes small or indistinct lauterbone organ. Ventromental plates fan-shaped and well seperated..(Chironomini) 6

5(4) Antennal lauterbon organ divided, more than 5x length of antennal segments 3–5 combined.. ***Tanytarsus kiseogi***

5′ Antennal lauterbon organ not divided, simple, as long as antennal segments 3–5 combined.. ***Tanytarsus ahyoni***

6(4′) Abdominal segment VII with lateral tubules; segment VIII with 2 pairs of ventral tubules 7

6′ Abdominal segment VII without lateral tubules; segment VIII with 1–2 pairs of ventral tubules 8

7(6) Mentum lateral teeth irregularly arranged .... ***Chironomus circumdatus***

7′ Mentum lateral teeth regularly arranged .... ***Chironomus nipponensis, Chironomus plumosus***

8(6′) Abdominal segment VIII with 1 pair of ventral tubules 9

8′ Abdominal segment VIII with 2 pair of ventral tubules 10

9(8) Mentum median tooth whole, not dentate; 4th lateral teeth smaller than 5th lateral teeth..................... .... ***Benthila carbonaria***

9′ Mentum median tooth with 4 small teeth; 4th lateral teeth same in size as 3rd or 5th lateral teeth .... ***Lipiniella moderata***

10(8′) Mentum with 1 median tooth.. 11

10′ Mentum with more than 2 median teeth 12

11(10) Median tooth somewhat depressed, lower than 1st lateral teeth .... ***Glyptotendipes tokunagai***

11′ Median tooth normal, as high as 1st lateral teeth .... ***Dicrotendipes nervous***

12(10′) Mentum median tooth double-forked; 1st lateral teeth smaller than 2nd lateral teeth .... ***Polypedilum yonsanensis***

12′ Mentum median tooth triple-forked; 1st lateral teeth similar in size to 2nd lateral teeth .... ***Microchironomus tener***
